# The histone methyltransferase DOT1L is required for proper DNA damage response, DNA repair, and modulates chemotherapy responsiveness

**DOI:** 10.1186/s13148-018-0601-1

**Published:** 2019-01-07

**Authors:** Vijayalakshmi Kari, Sanjay Kumar Raul, Jana Maria Henck, Julia Kitz, Frank Kramer, Robyn Laura Kosinsky, Nadine Übelmesser, Wael Yassin Mansour, Jessica Eggert, Melanie Spitzner, Zeynab Najafova, Holger Bastians, Marian Grade, Jochen Gaedcke, Florian Wegwitz, Steven A. Johnsen

**Affiliations:** 1 Department of General, Visceral and Pediatric Surgery, University Medical Center Göttingen, 37075 Göttingen, Germany; 2 Department of Biotechnology, Rama Devi Women’s University, Bhubaneswar, 751022 India; 3 Department of Pathology, University Medical Center Göttingen, 37075 Göttingen, Germany; 4 Department of Medical Statistics, University Medical Center Göttingen, 37075 Göttingen, Germany; 5 Department of Computer Science, University Augsburg, 86159 Augsburg, Germany; 6 Institute of Molecular Oncology, Section for Cellular Oncology, Göttingen Center for Molecular Biosciences (GZMB) and University Medical Center, University of Göttingen, 37077 Göttingen, Germany; 7 Laboratory of Radiobiology and Experimental Radiooncology, University Medical Center Hamburg-Eppendorf, 20246 Hamburg, Germany; 8 Tumor Biology Department, National Cancer Institute, Cairo University, Cairo, 11796 Egypt

**Keywords:** DOT1L, H3K79me, γH2AX, DNA damage, DNA double-strand breaks, Homologous recombination, PARP inhibition, FOLFIRI, Colorectal cancer

## Abstract

**Background:**

Disruptor of telomeric silencing 1-like (DOT1L) is a non-SET domain containing methyltransferase known to catalyze mono-, di-, and tri-methylation of histone 3 on lysine 79 (H3K79me). DOT1L-mediated H3K79me has been implicated in chromatin-associated functions including gene transcription, heterochromatin formation, and DNA repair. Recent studies have uncovered a role for DOT1L in the initiation and progression of leukemia and other solid tumors. The development and availability of small molecule inhibitors of DOT1L may provide new and unique therapeutic options for certain types or subgroups of cancer.

**Methods:**

In this study, we examined the role of DOT1L in DNA double-strand break (DSB) response and repair by depleting DOT1L using siRNA or inhibiting its methyltransferase activity using small molecule inhibitors in colorectal cancer cells. Cells were treated with different agents to induce DNA damage in DOT1L-depleted or -inhibited cells and analyzed for DNA repair efficiency and survival. Further, rectal cancer patient samples were analyzed for H3K79me3 levels in order to determine whether it may serve as a potential marker for personalized therapy.

**Results:**

Our results indicate that DOT1L is required for a proper DNA damage response following DNA double-strand breaks by regulating the phosphorylation of the variant histone H2AX (γH2AX) and repair via homologous recombination (HR). Importantly, we show that small molecule inhibitors of DOT1L combined with chemotherapeutic agents that are used to treat colorectal cancers show additive effects. Furthermore, examination of H3K79me3 levels in rectal cancer patients demonstrates that lower levels correlate with a poorer prognosis.

**Conclusions:**

In this study, we conclude that DOT1L plays an important role in an early DNA damage response and repair of DNA double-strand breaks via the HR pathway. Moreover, DOT1L inhibition leads to increased sensitivity to chemotherapeutic agents and PARP inhibition, which further highlights its potential clinical utility. Our results further suggest that H3K79me3 can be useful as a predictive and or prognostic marker for rectal cancer patients.

**Electronic supplementary material:**

The online version of this article (10.1186/s13148-018-0601-1) contains supplementary material, which is available to authorized users.

## Background

Colorectal cancer (CRC) is the third leading cause of cancer-related deaths worldwide [1]. The sequential accumulation of genetic mutations and epigenetic changes transforms the normal epithelia to adenoma and aggressive cancer [2]. Genomic instability is a hallmark of CRC, which is classified in major subtypes based on specific molecular signatures [3, 4]. Although recent advances in the treatment of CRC have increased patient survival, therapeutic resistance and relapse remain major challenges in treating CRC patients due to tumor heterogeneity and the development of resistance mechanisms [5].

DNA double-strand breaks (DSB) are the most lethal form of DNA damage, and unrepaired DSBs lead to chromosomal abnormalities and can result in a variety of pathological conditions including cancer [6]. In eukaryotes, the recognition and repair of DSBs is associated with post-translational histone modifications (PTMs) and requires chromatin remodeling and repair factors to enable proper recognition and repair of breaks and maintain genomic integrity [7]. One such modification is methylation of H3 at lysine 79 (H3K79me), mediated by the non-SET domain containing methyltransferase DOT1L, which catalyzes the mono-, di-, and tri-methylation of H3K79 [8, 9]. Several studies have demonstrated a role of Dot1 or its mammalian homolog DOT1L in mediating H3K79me and controlling genomic processes including gene transcription, splicing, telomeric silencing, and DNA damage repair [8, 10–14]. Further studies have implicated a role in heterochromatin maintenance and development [15]. Moreover, DOT1L interacts with the *MLL* translocation protein complex, thereby leading to aberrant methylation of target genes, and is associated with tumorigenesis and poor outcome [16–18]. Recently developed small molecule inhibitors of DOT1L are currently being tested in the treatment of MLL-rearranged leukemia [19–21]. We previously identified the *DOT1L* gene as 1 of 11 genes whose increased methylation is associated with better disease outcome in rectal cancer patients [22]. Though previous studies have suggested a role of DOT1L in DNA repair and transcription recovery after DNA damage, its role in DSB repair and the potential utility of DOT1L inhibitors in combination with standard of care therapies of CRC remain largely unknown.

In this study, we demonstrate the importance of DOT1L-mediated H3K79me3 in the early DNA damage response and the repair of DNA DSBs. Depletion or inhibition of DOT1L methyltransferase activity leads to an impaired DNA damage response indicated by decreased γH2AX levels, but increased phosphorylation of KAP1. Importantly, the loss of DOT1L function leads to defective HR-mediated DSB repair without affecting NHEJ. Importantly, loss of DOT1L or inhibition of its methyltransferase activity increased sensitivity to irradiation and chemotherapeutic agents used in the treatment of CRC patients. Consistent with the finding that defects in HR-mediated DSB repair lead to sensitivity toward poly (adenosine diphosphate [ADP]) ribose polymerase (PARP) inhibitors [23, 24], inhibition of DOT1L increased sensitivity to PARP inhibitors, further confirming its role in HR-mediated repair. Finally, by examining a cohort of rectal cancer patient samples, we provide the first evidence that patients with low H3K79me3 display a tendency toward overall poorer survival, indicating that this subgroup of patients with decreased H3K79me3 levels may benefit from treatment with PARP inhibitors.

## Results

### DOT1L is required for proper DNA damage response

**Fig. 1:**
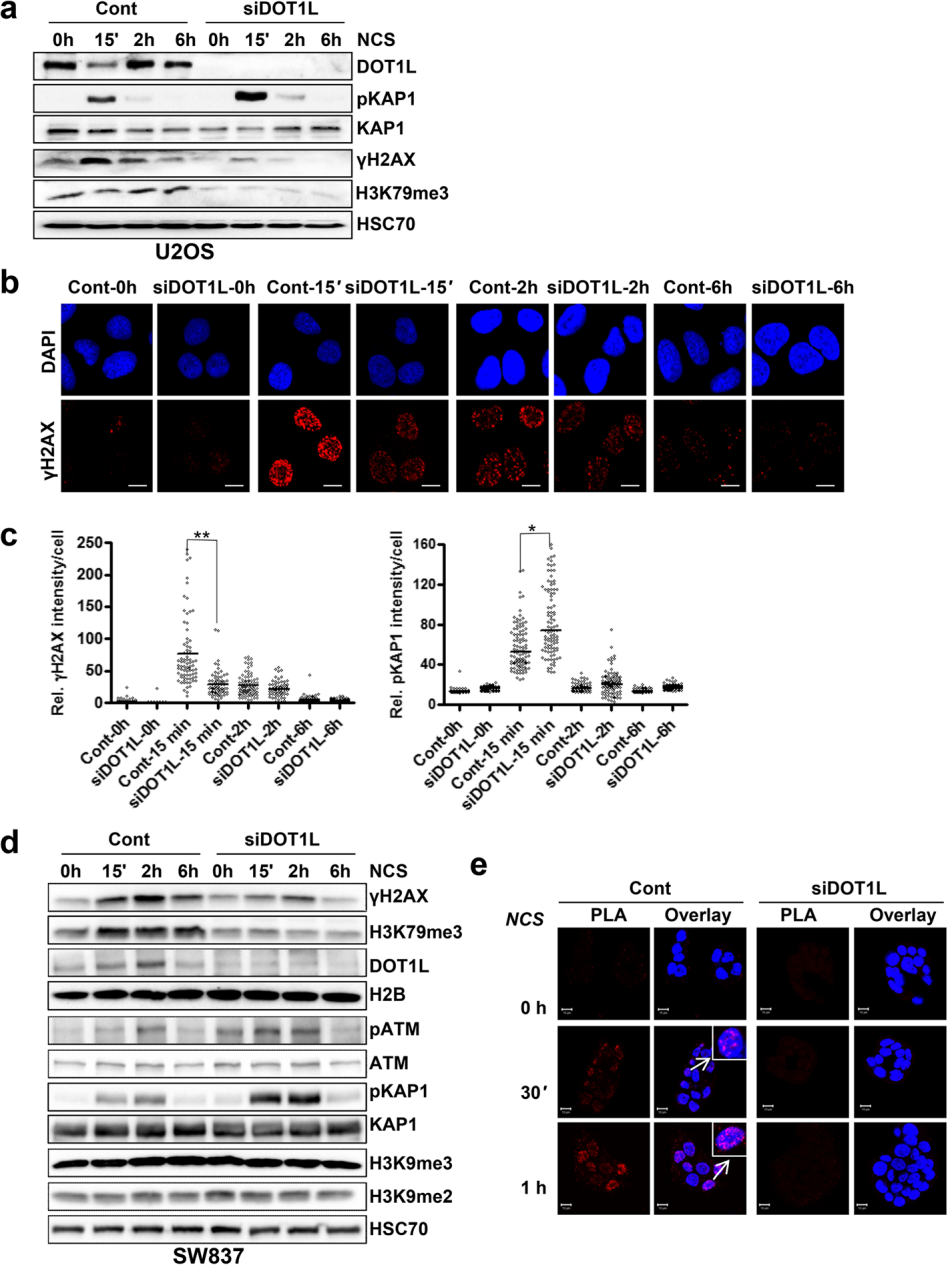
DOT1L is required for the proper DNA damage response. **a** U2OS cells were transfected either mock (Control—Cont) or with siRNA (smart pool of 4 siRNA—SP) for DOT1L. After 48 h of transfection, cells were treated with neocarzinostatin (NCS; 100 ng/ml) to induce DNA DSBs for the indicated time points. Total protein lysates were analyzed by Western blot for the indicated proteins. **b** Similar to **a**, U2OS cells were transfected with siRNA and after 48 h of transfection, cells were treated with NCS for the indicated time points and processed for immunofluorescence analysis for γH2AX in red and nuclei were stained with DAPI in blue (Scale bar—10 μM). **c** Quantification of γH2AX and pKAP1 levels in mock or DOT1L siRNA-transfected U2OS cells treated with NCS for the indicated times from b. More than 50 cells were measured for each condition and represented as relative intensity in each cell, mean values are represented in the plot *n* = 3, ± SD, *p* values (**0.005 and *0.01). **d** SW837 cells were either mock (Cont) transfected or with DOT1L siRNA, after 72 h cells were treated with NCS (100 ng/ml) to induce DSBs and total protein lysates were immunoblotted with the indicated antibodies. **e** Proximity ligation assay (PLA) for γH2AX and H3K79me3 in SW837 cells transfected with either mock (Cont) or DOT1L siRNA for 72 h prior to treatment with NCS (100 ng/ml) for the indicated time points before processing for PLA. Red dots indicate regions of close proximity between γH2AX and H3K79me3. Nuclei were stained with DAPI (Scale bar—10 μM)

Phosphorylation of H2AX at serine 139 (γH2AX) by specific DNA damage response-associated members of the phosphatidylinositol-3-kinase family is an early marker of DNA damage induction. In order to examine a potential role of DOT1L in the DNA damage response to DNA double-strand breaks (DSB), initially DOT1L was efficiently depleted in U2OS osteosarcoma cells, a cell line widely used to study DNA repair mechanisms, and DSBs were induced by the radiomimetic drug neocarzinostatin (NCS). Western blot analysis with total protein lysates for γH2AX demonstrated increased γH2AX within 15 min of NCS treatment which decreased to basal levels by 6 h, consistent with a near complete repair of DSBs. Interestingly, DOT1L-depleted cells showed only a moderate increase in the levels of γH2AX 15 min after DSB induction, suggesting that DOT1L depletion may compromise the early DNA damage response (Fig. 1a). Moreover, no further increase in γH2AX was observed at any of the time points analyzed. Given the fact that DOT1L methylates H3K79, we examined global H3K79me3 levels in both control cells and following DSB induction. In contrast to γH2AX, H3K79me3 levels were only moderately increased following DSB induction with significantly slower kinetics (Fig. 1a). As expected, H3K79me3 levels were significantly decreased in DOT1L-depleted cells (Fig. 1a). Moreover, we also observed increased phosphorylation of KAP1 at serine 824 (pKAP1) in DOT1L-depleted cells compared to control cells 15 min after DSB induction (Fig. 1a). Immunofluorescence studies using U2OS cells further confirmed that γH2AX induction is compromised, while pKAP1 levels were elevated in DOT1L-depleted cells (Fig. 1b, Additional file 1: Figure S1c). The quantification of γH2AX and pKAP1 levels from control and DOT1L-depleted cells confirm that the overall intensity of γH2AX is decreased, while pKAP1 levels were elevated in DOT1L-depleted cells compared to control cells 15 min after DNA DSB induction (Fig. 1c) and the effects were consistent over time. Similar results were observed in the rectal cancer cell line SW837, where the depletion of DOT1L (Additional file 1: Figure S1a, b) also led to decreased induction of γH2AX 15 min after DSB induction (Fig. 1d), while pKAP1 levels were elevated compared to control cells (Fig. 1d, Additional file 1: Figure S1d). We also observed some variation in DOT1L levels following DSB induction (Fig. 1a, d); however, these effects were highly variable between cell lines and experiments. Next, we analyzed the activation of ataxia telangiectasia mutated kinase (ATM), an upstream kinase for H2AX, which is auto-phosphorylated at serine 1981 upon DSB induction. Interestingly, DOT1L-depleted cells displayed slightly elevated levels of phosphorylated ATM compared to control cells upon DSB induction, suggesting that the observed decrease in γH2AX levels following DOT1L depletion was not due to defective upstream kinase activity (Fig. 1d, Additional file 1: Figure S1e). This further suggests that DOT1L or DOT1L-mediated H3K79 methylation controls the early DNA damage response pathway.

Since phosphorylation of KAP1 is associated with DNA repair in heterochromatic regions, we also examined whether DOT1L depletion may elicit global changes in heterochromatin markers. However, no changes in H3K9me2, H3K9me3, or HP1 levels were observed in DOT1L-depleted cells (Fig. 1d, Additional file 1: Figure S1g), suggesting that the observed effects on KAP1 phosphorylation are not simply due to a global re-partitioning of euchromatin into heterochromatin. Since earlier studies indicated that DOT1L or DOT1L-mediated H3K79me may play an important role in the early DNA damage response, we used the proximity ligation assay (PLA) to examine whether H3K79me3 and γH2AX co-localize upon DSB induction. Indeed, while global H3K79me3 levels were unchanged following DSB induction, γH2AX and H3K79me3 were co-localized within 30 min following DSB induction and a further increase was observed at 1 h after DSB induction. The specificity of the assay was confirmed by DOT1L depletion, which led to decreased H3K79me3 levels and a loss of PLA signal (Fig. 1e). The co-localization of γH2AX and 53BP1 served as a positive control for the experiment (Additional file 1: Figure S1f, g).

### DOT1L is required for homologous recombination-mediated DSB repair

**Fig. 2:**
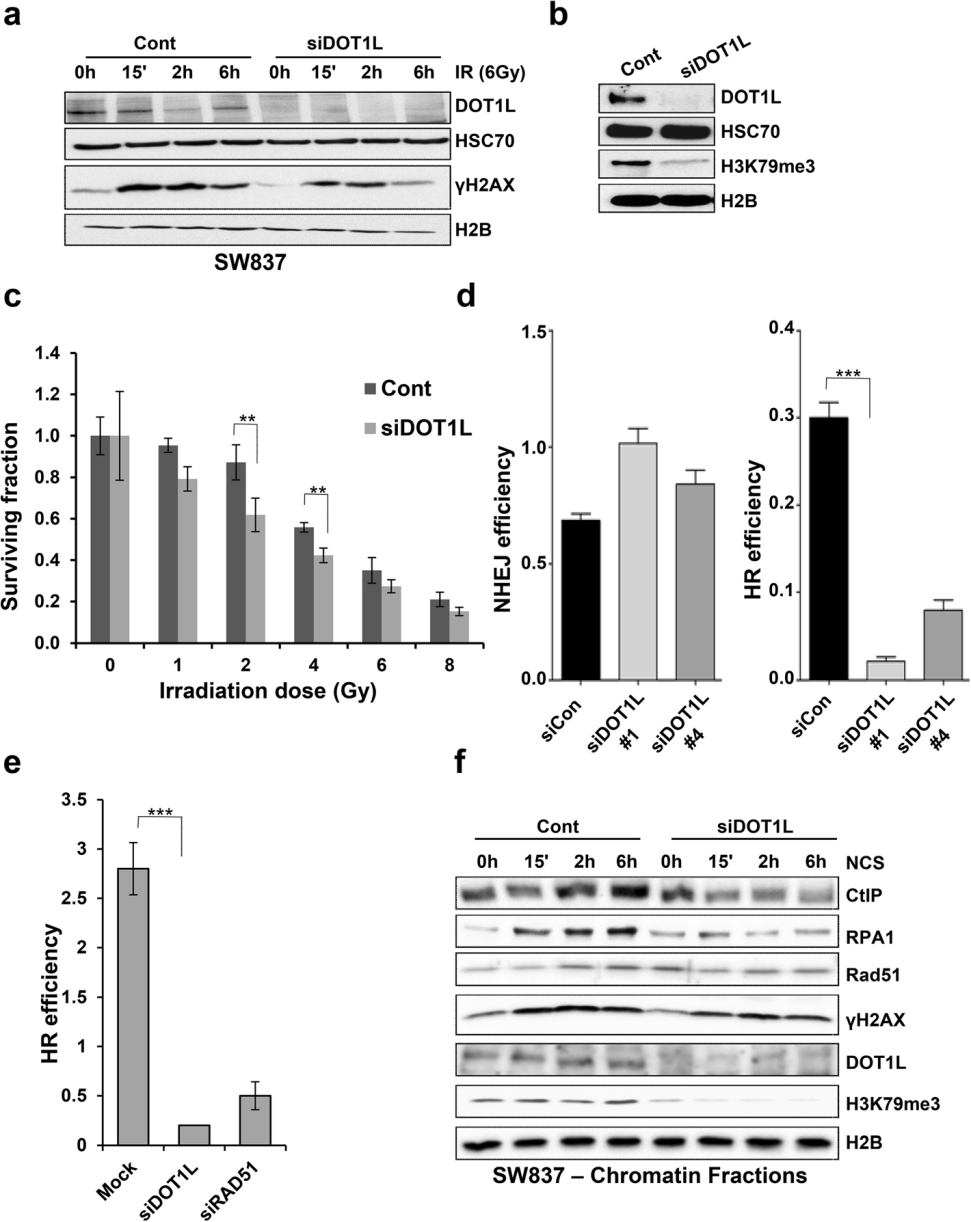
DOT1L is required for homologous recombination-mediated DNA DSB repair. **a** SW837 cells were transfected either mock or with DOT1L siRNA (SmartPool) for 48 h, treated with ionizing radiation (6 Gy), and total protein lysates were harvested at the indicated time points and immunoblotted with the indicated antibodies. H2B and HSC70 were used as loading controls. **b** DOT1L knockdown efficiency in siRNA-transfected SW837 cells (parallel to **a**) was verified in whole cell lysates with DOT1L and H3K79me3 antibodies. **c** Colony formation assays were performed with control and DOT1L-depleted SW837 cells from b with the indicated doses of irradiation (Gy) and data represent the mean values from the surviving fraction (SF), *n* = 3, ± SD, *p*-value (0.005–4 Gy). **d** HeLa cells harboring single copies of an NHEJ repair substrate (pEJ) or an HR repair substrate (pGC) were transfected with either mock or DOT1L siRNA #1 or #4 and a DSB was induced by transfecting cells with an I-SceI-expression vector (pCMV-I-SceI-3xNLS). After 48 h, the percentage of GFP-positive cells was measured using flow cytometry analysis as an indication for HR and NHEJ efficiency. **e** HCT116 cells with an integrated GFP reporter system for HR efficiency were transfected with the indicated siRNAs and GFP-positive cells were measured using flow cytometry analysis. **f** SW837 cells were transfected as in b and 72 h after transfection treated with NCS (100 ng/ml) for the indicated time points. Chromatin fractions were harvested and subjected to Western blot analysis for the indicated proteins

Based on our results indicating that DOT1L is required for the early DNA damage response, we further investigated a role for DOT1L in DSB repair. Initially, control and DOT1L-depleted SW837 cells were irradiated with 6 Gy to induce DSBs and total protein lysates were analyzed by Western blot analysis. Similar to our results using NCS, DOT1L depletion resulted in decreased induction of γH2AX compared to control cells (Fig. 2a). We further analyzed the sensitivity of DOT1L-depleted cells toward irradiation (Fig. 2b). Colony formation assays confirmed that DOT1L-depleted cells were more sensitive to irradiation compared to the control cells, suggesting that DOT1L may be required for DSB repair (Fig. 2c). Since DSBs are repaired by two major pathways, namely by the error prone non-homologous end joining (NHEJ) and error-free homologous recombination (HR) pathways [25], we next sought to determine whether DOT1L preferentially promotes repair of DSBs by one or both of these pathways. Therefore, DOT1L was depleted in HeLa cells harboring single copies of a plasmid-based GFP-reporter specific for NHEJ- or HR-mediated DNA DSB repair (pEJ and pGC, respectively). This system allows for a precise measurement of the efficiency of NHEJ and HR based on the number of GFP^+^ cells measured by flow cytometry. Interestingly, depletion of DOT1L using two different siRNAs led to decreased HR-mediated repair without impairing NHEJ-mediated repair (Fig. 2d). Importantly, these findings could be confirmed in HCT116 cells harboring the stably integrated HR substrate (Fig. 2e and Additional file 2: Figure S2a). To further validate the role of DOT1L in HR-mediated DSB repair, we performed Western blot analysis of chromatin fractions from control and DOT1L-depleted SW837 cells after DSB induction. Repair of DSBs via HR is particularly dependent upon the resection of one strand of DNA at the break sites. This process is controlled by many proteins where, in particular, CtBP-interacting protein (CtIP) is recruited at an early stage of the end resection process [26]. Our results show that while CtIP is recruited to chromatin in control cells following DSB induction; this recruitment is severely compromised in DOT1L-depleted cells, indicating that DNA end resection is defective in the absence of DOT1L (Fig. 2f). End resection generates a stretch of 3′ single-stranded DNA (ssDNA) that is coated with the single-stranded DNA (ssDNA) binding proteins RPA1 and RAD51. Since we observed decreased CtIP recruitment in DOT1L-depleted cells, we next examined the binding of RPA1 and RAD51 proteins to the chromatin as an indicator of end resection processing and ssDNA generation. Consistent with our findings that CtIP recruitment was impaired following DOT1L depletion, we also observed decreased recruitment of RPA1 and RAD51 to DNA in DOT1L-depleted cells (Fig. 2f). Consistently, RAD51 phosphorylation was also decreased in DOT1L-depleted cells (Additional file 2: Figure S2b) and the effects were not due to changes in cell cycle regulation upon DOT1L depletion (Additional file 2: Figure S2c). Thus, together, these findings indicate that DOT1L is required specifically in the end resection step of HR-mediated DSB repair.

### Inhibition of DOT1L leads to increased sensitivity to DNA damaging agents

**Fig. 3:**
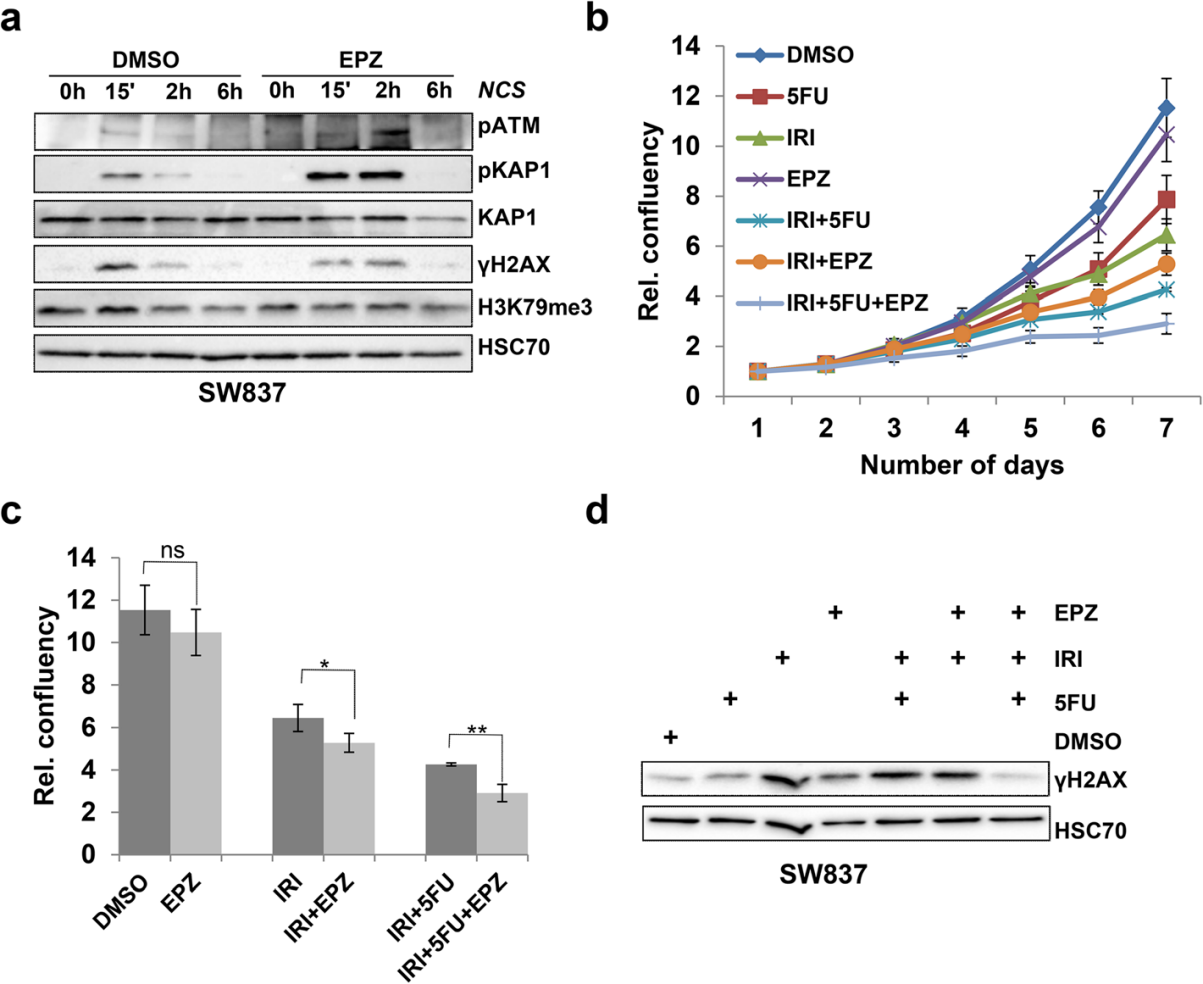
Inhibition of DOT1L leads to increased sensitivity to DNA damaging agents. **a** SW837 cells were pretreated with either with DMSO or the DOT1L inhibitor EPZ5676 (1 μM) for 3 h followed by treatment with NCS (100 ng/ml) for the indicated time points. Whole cell lysates were immunoblotted and detected with the indicated antibodies. **b** Cell proliferation assay with SW837 cells treated either with 5-fluorouracil (5-FU; 5 μM), Irinotecan (IRI; 1 μM), EPZ5676 (1 μM) individually or in combination as indicated. Cell confluency was measured every 24 h using a Celigo cytometer. **c** Bar graph representation of cell proliferation analysis from **b** at day 7. Data are represented as mean values *n* = 3, ± SD, and *p* values (ns, *0.01, **0.003). **d** Western blot analysis from SW837 cells treated as in **b** for 48 h with the indicated compounds

We next sought to determine whether pharmacological inhibition of DOT1L methyltransferase activity, using the recently developed small molecular inhibitor EPZ5676 (EPZ; also called pinometostat) [19, 27], which was tested in phase I clinical trials for adult and pediatric MLL-translocated leukemia (NCT01684150 and NCT02141828, respectively) [28], also impairs the DNA damage response and repair. Thus, DOT1L activity was inhibited by EPZ treatment of SW837 cells for either 3 h (acute inhibition) or 72 h (prolonged inhibition) followed by treatment with NCS to induce DSBs. Consistent with siRNA-mediated DOT1L depletion, Western blot analysis of total protein lysates revealed that both acute and prolonged DOT1L inhibition result in decreased accumulation of γH2AX (Additional file 3: Figure S3a). Notably, a decrease in global H3K79me3 was only observed after prolonged (72 h) EPZ treatment, but relatively unaffected within 3 h (Additional file 3: Figure S3a, Fig. 3a), suggesting that DSB-induced DOT1L activity at sites of DNA damage, rather than pre-existing H3K79me3, may be important for HR-mediated DNA repair. Similar results were observed using another chemically distinct DOT1L inhibitor SGC094a, where treatment for 3 or 72 h similarly led to decreased γH2AX levels following NCS treatment (Additional file 3: Figure S3b). Moreover, phospho-ATM and phospho-KAP1 levels were also increased following DOT1L inhibition for 3 h (Fig. 3a). Together, these findings demonstrate that DOT1L methyltransferase activity is specifically required for a proper DNA damage response.

Given the potential clinical application of DOT1L inhibitors in cancer treatment, we next tested the effects of combinatorial treatment with standard of care therapies for colorectal cancer, where 5-fluorouracil (5-FU), a nucleoside analogue, is frequently combined with the clinically utilized topoisomerase I inhibitor irinotecan (IRI) in a combination referred to as FOLFIRI [29, 30]. Thus, we tested how treatment with the clinical DOT1L inhibitor EPZ5676 would impact the effects of the individual and combined treatments with IRI and 5-FU on cell proliferation and survival. As expected, individual treatments with IRI or 5-FU alone decreased the proliferation of SW837 cells and combination of the two treatments further decreased cell proliferation. In contrast, EPZ treatment alone had very little effect on cell proliferation, but enhanced the anti-proliferative effects of 5-FU, IRI, and combined treatment (Fig. 3b, c). The observed effects are likely due to an impaired DNA damage response since global γH2AX levels were increased by IRI, 5-FU, and combined IRI/5-FU 48 h after treatment, while the combination of IRI/5-FU and EPZ resulted in decreased γH2AX levels compared to IRI, 5-FU, or IRI/5-FU (Fig. 3d).

### Inhibition of DOT1L leads to increased sensitivity to PARP inhibition

**Fig. 4:**
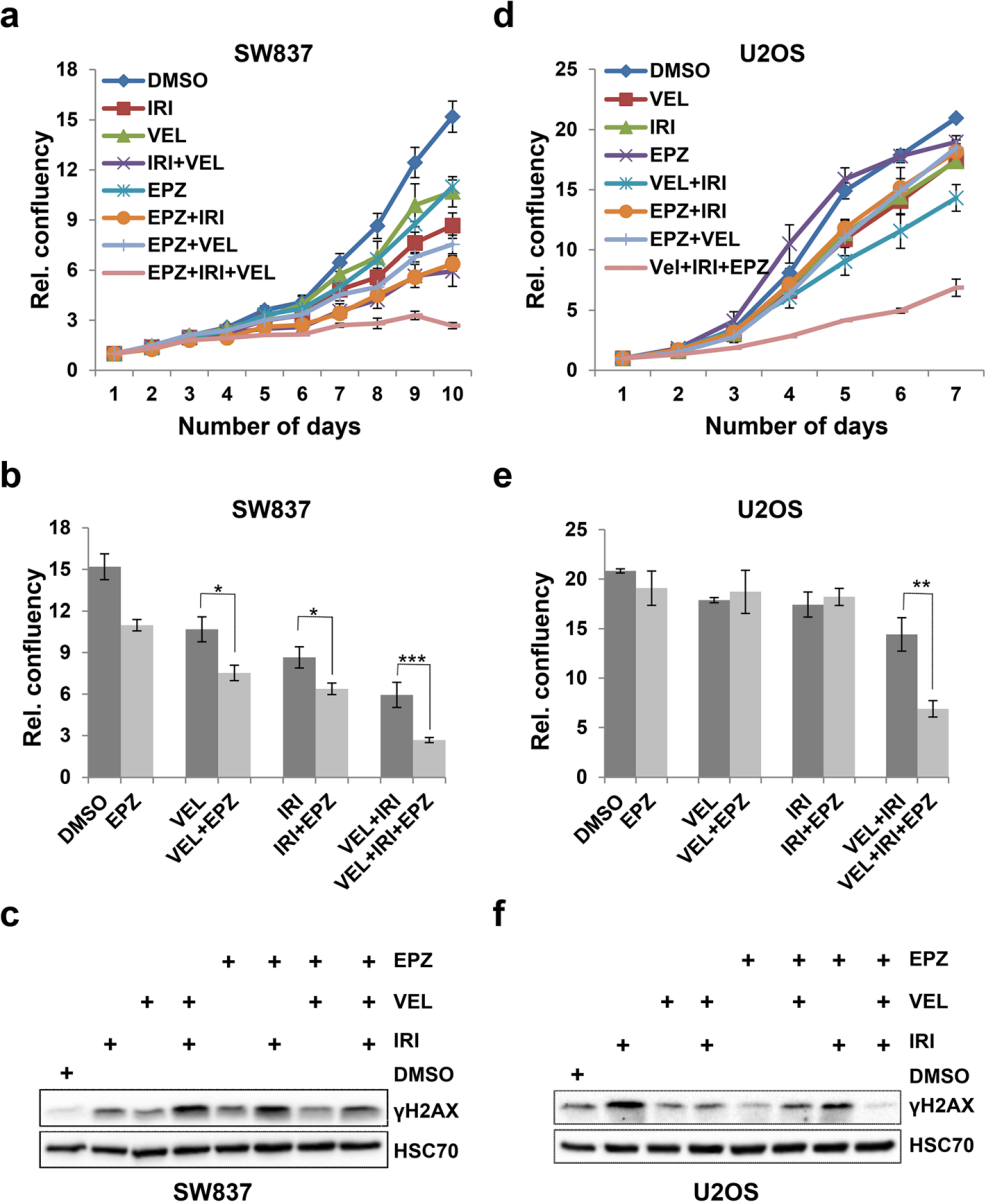
Inhibition of DOT1L leads to increased sensitivity to PARP inhibition. **a**, **d** Cell proliferation of SW837 (**a**) and U2OS (**d**) cells treated either with IRI (1 μM), EPZ (1 μM), or Veliparib (VEL) individually or in combination and measured every 24 h using the Celigo cytometer. **b**, **e** Bar graph representation of cell proliferation of SW837 cells from **a** at day 10 and U2OS cells from D at day 7. Data are represented as mean values *n* = 3, ± SD, and *p* value (**b** *0.01, *0.03, ***0.0001; **e** **0.002). **c**, **f** Western blot analysis from SW837 (**c**) and U2OS (**f**) cells treated as in (**a**, **d**) after 48 h of treatment. Total protein lysates were harvested and analyzed by Western blot using antibodies against the indicated proteins

A unique and therapeutically useful feature of tumor cells with HR deficiencies (e.g., *BRCA1* mutations) is an increased sensitivity to PARP inhibitors [23, 31]. Since we specifically observed defective HR-mediated DSB repair in DOT1L-depleted cells and following inhibition of DOT1L methyltransferase activity, we hypothesized that loss of DOT1L activity may lead to sensitivity to PARP inhibitors. In order to test this, we treated SW837 cells with a DOT1L inhibitor (EPZ), IRI, the PARP inhibitor Veliparib (VEL), or combinations thereof. Consistent with our hypothesis, inhibition of DOT1L methyltransferase activity led to increased sensitivity toward IRI or VEL, with the combination of all three agents having the greatest effect on SW837 rectal carcinoma cells (Fig. 4a, b). Similar results were observed with U2OS cells, where the combination of EPZ, IRI, and VEL also decreased the proliferation of cells more than the individual treatments (Fig. 4d, e). Consistent with the effects observed with NCS, irradiation, and IRI/5-FU treatments, activation of the DNA damage response (e.g., γH2AX levels) was decreased following the combination treatment in both cell lines (Fig. 4c, f).

### Patients with low H3K79me3 levels show poorer overall survival

**Fig. 5:**
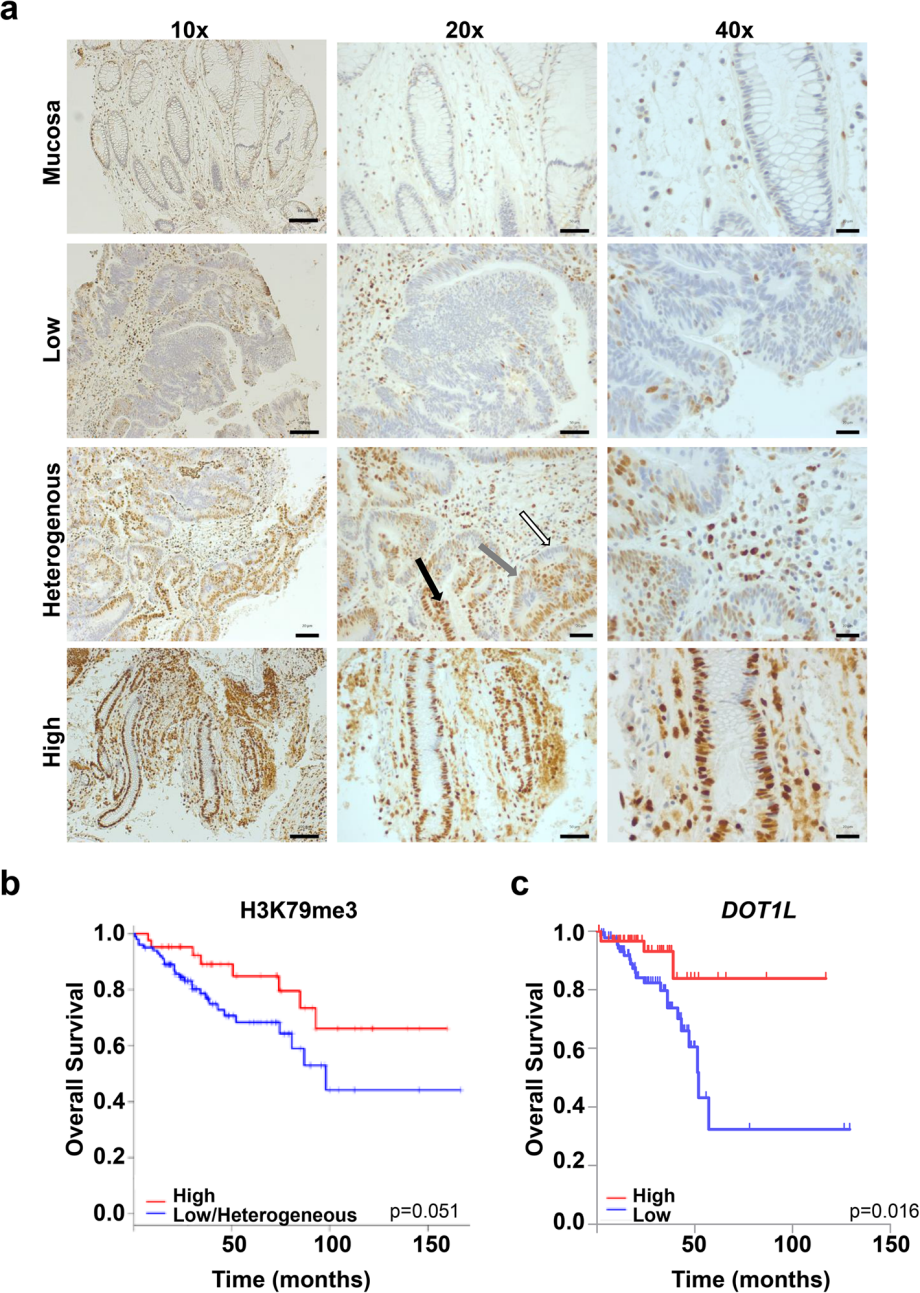
Patients with low H3K79me3 or DOT1L mRNA levels show poorer overall survival. **a**, **b** In total, 156 rectal cancer patient biopsy samples from treatment naïve patients were analyzed by immunohistochemistry for H3K79me3. Based on the staining intensity, the nuclei were given the relative score of 0 to 3, with 3 corresponding to highest staining intensity. Moreover, the overall percentage of the stained nuclei corresponding to each score was estimated for each analyzed sample. **a** Patient tissue samples were then classified based on the staining intensity and overall percentage of the stained nuclei as no staining, or low (5% of the nuclei with low score), medium/heterogeneous (50% of the nuclei stained heterogeneously), or high (100% of the nuclei with high score) staining. Solid arrows indicate positive, gray arrows weak, and white arrows negative staining. **b** The Kaplan-Meier survival plot for groups of patients with tissue samples classified as “High” (red line) and “Low or Heterogeneous” (blue line; comprising patients with no, low or heterogeneous staining) (*p* value, 0.051). **c** Separation of rectal adenocarcinoma patients from The Cancer Genome Atlas into “high” (*n* = 67) and “low” (*n* = 92) *DOT1L* mRNA expression (greater than or less than 4.7 reads per kilobase per million, respectively) displayed in the Kaplan-Meier survival plot revealed better overall survival (*p* value, 0.018) in patients with higher levels of *DOT1L* expression

Defects in HR-mediated DNA repair are frequently associated with poorer patient survival. Thus, we hypothesized that DOT1L-mediated H3K79me3 levels may correlate with patient outcome in colorectal cancer. In order to test this, we performed immunohistochemistry analysis for H3K79me3 on tumor microarrays (TMAs) derived from treatment naïve rectal cancer patients (*n* = 156). Interestingly, while normal mucosa consistently displayed low levels of H3K79me3, tumors displayed varying levels of H3K79me3, with many displaying heterogeneous staining within the tumor (Fig. 5a). Notably, low or heterogeneous H3K79me3 levels were associated with an overall poorer prognosis compared to patients with tumors displaying higher levels of H3K79me3 showing a better overall survival (Fig. 5b). Consistent with these findings, analysis of *DOT1L* mRNA levels in rectal adenocarcinoma patient gene expression data from the TCGA research network (http://cancergenome.nih.gov/) revealed a similar trend with patients displaying higher *DOT1L* expression having a significantly better overall survival compared to patients with lower *DOT1L* expression levels (Fig. 5c).

## Discussion

Proper DNA damage recognition and response are key steps necessary to repair DSBs in a timely manner and to prevent genomic instability and the development of cancer. Histone modifications and chromatin remodelers play an important role in repairing DNA damage, especially at DSBs [6, 7]. In this study, we elucidated a function of the H3K79 histone methyltransferase DOT1L in controlling the DNA damage response and repair of DSBs.

Our results show that depletion of DOT1L impaired the DNA damage response as assessed by the accumulation of γH2AX at 15 min following DSB induction. This indicates that DOT1L plays an essential central role in the early DNA damage response, while the activation of the upstream kinase ATM (as assessed by autophosphorylation of Ser1981) was not inhibited by DOT1L depletion or inhibition. Interestingly, DOT1L depletion or inhibition resulted in increased accumulation of phosphorylated KAP1, a protein associated with DNA repair at heterochromatic regions, indicating that DOT1L likely also influences DSB repair in heterochromatin [32, 33]. Since we did not observe global changes in the heterochromatin markers H3K9me2/3, this effect does not appear to be due to global changes in chromatin compartmentalization. Importantly, these effects could be observed after acute treatment (3 h) with the DOT1L methyltransferase inhibitor EPZ5676, which did not cause a global loss of H3K79me3, suggesting that DOT1L likely plays a direct methyltransferase-dependent function at DSBs, rather than affecting DSB repair via the transcription-associated marking of active chromatin regions. This finding is consistent with our proximity-ligation assays demonstrating a co-localization of H3K79me3 with γH2AX after DSB induction.

Consistent with a role of DOT1L in DNA repair processes, our results show that DOT1L-depleted cells are more sensitive to ionizing irradiation. Plasmid-based GFP-reporter assays suggest that this effect is likely due to impaired HR-, but not NHEJ-mediated DSB repair. This finding is supported by the observation that recruitment of CtIP, a protein critical in the DNA end resection process during HR-mediated repair, as well as the subsequent loading of the ssDNA binding proteins RPA1 and RAD51 to chromatin is impaired in the absence of DOT1L activity. Thus, DOT1L may cooperate with other chromatin remodeling factors such as CHD1 to specifically open chromatin to promote the DNA end resection process [34].

The DOT1L inhibitor Pinometostat (EPZ5767) used in this study recently completed a phase I clinical trial for adult leukemia (NCT01684150) and was shown to have modest activity as a single agent, but was generally safe and, therefore, potentially suitable for combinatory approaches with other chemotherapeutic agents [28]. Indeed, our study suggests that combining EPZ with standard of care treatment for colorectal cancer (FOLFIRI) can significantly increase therapeutic efficacy. This effect is likely mechanistically linked to the central role of DOT1L in HR-mediated DNA repair. Consistently, in much the same way that the combination of EPZ, VEL, and IRI significantly decreased cell survival compared to the individual treatments, we also showed that inhibition of DOT1L activity also increased sensitivity towards PARP inhibitors.

Our previous work identified the *DOT1L* gene as a differentially methylated gene in rectal cancer patients whose methylation levels in pre-therapeutic rectal cancer biopsies was associated with a better outcome [22]. Interestingly, in this study, no correlation between gene expression and DNA methylation could be observed, possibly implying that the expression of *DOT1L* following therapeutic challenge (rather than prior to treatment) may be important for enabling tumor cells to respond to therapeutic insult. In this study, we built upon these findings and examined whether rectal cancer patients may potentially be classified based on H3K79me3 levels or DOT1L mRNA levels. Indeed, when patient samples were classified into two major categories having either low or heterogeneous versus high H3K79me3 levels, we observed that low/heterogeneous H3K79me3 levels correlated with a poorer patient prognosis. Consistently, higher *DOT1L* mRNA levels also correlated with a better overall survival. Thus, H3K79me3 levels and/or DOT1L mRNA levels may potentially serve as prognostic markers, and potentially predict differential responsiveness to different standard of care treatments as well as sensitivity to PARP inhibitors.

## Conclusion

In this study, we show that DOT1L is required for the proper DNA damage response following DSBs induction. Moreover, cells with decreased DOT1L activity show hypersensitivity to irradiation and display defects in HR-mediated DNA repair. Consistently, small molecule inhibition of DOT1L methyltransferase increases sensitivity to chemotherapeutic agents such as 5-FU, irinotecan, and PARP inhibitor, suggesting its potential as a therapeutic target in rectal cancer. Future studies will be needed to further test the in vivo therapeutic efficacy of these findings and determine whether a combined inhibitor approach and/or H3K79me3-/DOT1L-based patient stratification may serve to improve patient outcome.

## Methods

### Cell culture and siRNA transfections

U2OS, SW837, and HCT116 cells were cultured in Dulbecco’s modified Eagle’s medium (DMEM) (Invitrogen, DMEM/F12, and Roswell Park Memorial Institute medium (RPMI 1640)) respectively, supplemented with 10% fetal bovine serum (FBS) and 1x penicillin–streptomycin (Sigma, St. Louis, USA) at 37 °C under humidified condition with 5% CO_2_. HeLa cells harboring pEJ or pGC substrates were grown in DMEM medium containing 800 μg/ml G418 or 1 μg/ml puromycin, respectively. For transient depletion of DOT1L, siRNA transfections (sequences in Table 1) were performed using Lipofectamine RNAiMAX (Invitrogen, Waltham, USA) according to the manufacturer’s instructions. Primer sequences for the verification of depletion of DOT1L mRNA levels are indicated in Table 2. For DOT1L transfections, we used SmartPool (Dharmacon) of four siRNAs (Table 1) or the indicated individual siRNAs. After 72 h of transfection, DNA double-strand breaks were either induced with neocarzinostatin (NCS, N9162, Sigma, 100 ng/ml) or with ionizing radiation as indicated. To inhibit the methyltransferase activity of DOT1L, cells were treated with either EPZ5676 (S7062; Selleckchem) or with SGC094 (SML1107, Sigma) as indicated or in combination with 5-fluorouracil (5-FU, F6627; Sigma), Veliparib (ABT-888, S1004; Selleckchem), or irinotecan (I1406; Sigma) (Table 2).

**Table 1:** List of siRNA used in this study

Gene	Sequence 5′ to 3′
siDOT1L-1	CGAAGUGGAUGAAAUGGUA
siDOT1L-2	CCGAGAAGCUCAACAACUA
siDOT1L-3	GAAGCCGUCUCCCUCCAAA
siDOT1L-4	GAAACAAGCUCUAGAUCAU
Luciferase GL2 duplex	GCAGAAUCGUGUCCUCGAA
siGENOME non-targeting siRNA pool # 1	D-001206-13-05 (Dharmacon)

**Table 2:** RT-PCR primers used in this study

Name	Sequence	Source
h DOT1L For	CCACCAACTGCAAACATCAC	This study
h DOT1L Rev	AGAGGAAATCGCCTCTCTCC	This study
HNRNPK For	ATCCGCCCCTGAACGCCCAT	[36]
HNRNPK Rev	ACATACCGCTCGGGGCCACT	[36]

### Immunofluorescence

Cells were grown on sterile coverslips and transfected with the indicated siRNAs. After 72 h of transfection, the cells were treated with neocarzinostatin (NCS; N9162, Sigma-Aldrich) as indicated. Cells were fixed with 4% paraformaldehyde for 10 min at room temperature and then permeabilized with 0.1% Triton X-100 in PBS for 5 min. Cells were blocked with 3% bovine serum albumin (BSA) in PBS and incubated overnight with primary antibody at 4 °C in 3% BSA. Cells were incubated with fluorescent tagged secondary antibodies in 3% BSA for 1 h at room temperature and the nuclei were counterstained with 4′-6-diamidino-2-phenylindole (DAPI, 10 ng/ml). Mounted slides were examined and images were acquired using a Zeiss LSM 510 Meta confocal microscope using 25X or 63X oil immersion lens. For the quantification of immunofluorescence signal, more than 50 cells were quantified using ImageJ software from three biological replicates and represented as mean ± SD. *P* values were calculated by ANOVA method (*P ≤ 0.05, **P ≤ 0.01, and ***P ≤ 0.001).

### Immunohistochemistry and analysis of patient samples

Immunohistochemistry was performed using rectal adenocarcinoma tissue microarrays. Paraffin-embedded sections (2 μm) were incubated in 100% xylene for 20 min, followed by rehydration in descending dilutions of EtOH series (100%, 90%, and 70%) before washing with 1X PBS. Slides were an antigen retrieval buffer (citrate buffer pH 6, 0.05% Tween 20) for 3 min at 100 °C. Tissue sections were allowed to cool to room temperature and washed with PBS three times, quenched for endogenous peroxidase activity with 3% hydrogen peroxide (H_2_O_2_) treatment for 10 min at RT, and then washed three times with PBS. Afterward, sections were blocked using SEA BLOCK blocking buffer for 20 min at RT followed by the primary antibody for H3K79me3 (1:250) overnight at 4 °C. Sections were washed three times using PBS before adding the biotinylated secondary antibody (1:200, Envision Goat-anti-rabbit, Dako, Hamburg) and incubating for 1 h at RT. Sections were washed three times with PBS followed by Avidin-Peroxidase incubation diluted 1:1000 for 45 min. Signals were detected using diaminobenzidine (DAB) substrate (Dako, Hamburg) for 8 min at RT. Slides were washed and hematoxylin (Mayer’s hemalaun solution) was used for counterstaining for 5 min. Histological slides were imaged using an Axioscop microscope and ZEN software (Carl Zeiss, Jena, Germany). Publically available gene expression data from The Cancer Genome Atlas consortium were evaluated for *DOT1L* expression using the Protein Atlas (https://www.proteinatlas.org/) tool. Patients displaying “high” or “low” levels of *DOT1L* expression were defined using the cutoff of 4.7 reads per million per kilobase (RPKM).

### SDS-PAGE and Western blot analyses

Total cell protein lysates were prepared by lysing cells in RIPA (radio immunoprecipitation buffer) containing 1X PBS, 1% NP-40, 0.5% sodium deoxycholate, 0.1% SDS, 1 mM Pefabloc, 1 ng/μL Aprotinin/Leupeptin, 10 mM β-glycerophosphate, and 1 mM *N*-ethylmaleimide. The lysates were resolved by SDS-PAGE and blotted to nitrocellulose membranes and then blocked with either 3% BSA or 5% non-fat milk. The membranes were incubated with primary antibodies overnight at 4 °C followed by incubation with secondary antibodies coupled to horseradish peroxidase at RT. The membranes were developed using enhanced chemiluminescence and images were obtained using a ChemiDoc™ XRS+ System (Bio-Rad, California, USA). The list of antibodies used in this study is listed in Table 3.

**Table 3:** List of antibodies used in this study

Name	Cat. No	Company	Application
γH2AX	05-636	Millipore	WB, IF
53BP1	sc-22760	Santa Cruz	WB, IF
ATM	A1106	Sigma	WB
pATM (Ser1981)	4526	Cell Signaling	WB
CtIP	61141	Active Motif	WB
DOT1L	A310-953A	Bethyl Laboratories	WB
H2B	ab19847	Abcam	WB
H3	ab10799	Abcam	WB
H3K79me3	C15410068 (pAb-068)	Diagenode	WB, IF
H3K9me3		Diagenode	WB
H3K9me2	mAb154-050	Diagenode	WB
HSC70	sc-7298	Santa Cruz	WB
KAP1	A300-274A	Bethyl Laboratories	WB
pKAP1 (S824)	A300-767A	Bethyl Laboratories	WB, IF
RAD51	sc-8349	Santa Cruz	WB
pRAD51	ab111568	Abcam	WB
RPA1	NA13	Calbiochem	WB

### Analysis of DSB repair by GFP-based reporter assay

To measure the repair efficiency of DNA double-strand breaks, DSB reporter assays were used as described [34]. To measure the HR and NHEJ efficiency, HeLa cells containing the stably integrated reporter construct pGC for HR and pEJ for NHEJ were transfected with DOT1L siRNA 1 or 4. After 48 h of transfection, cell were transfected with the I-SceI endonuclease expression vector (pCMV3xnls-I-SceI, a kind gift from M. Jasin) to induce DSBs and pCMV-Neo as a control vector. After 24 h of DSB induction, cells were analyzed by flow cytometry (FACScan, BD Bioscience) for GFP-positive cells as an indication of HR and NHEJ efficiency. Data were normalized to transfection efficiency and represented as mean ± SD (*n* = 3). For HR assay, HCT116 cells harboring stably integrated pHPRT-pDRGFP plasmid were transfected with the indicated siRNAs. After 24 h of siRNA transfections, cells were transfected with pCBASceI (Addgene) to induce DSBs and pEGFP as a positive control by electroporation. To analyze HR efficiency, GFP expression was quantified by flow cytometry using BD FACS Canto II (BD FACSDiva™ 6.1.3, BD Biosciences) after 48 h of transfection. Additional information about flow cytometric analysis is provided in Additional file 4: supplementary methods.

### Cell proliferation assay

For cell proliferation assay, approximately 1000–2000 cells were seeded in 96-well plates per well. After 24 h, cells were treated with individual or combinations of compounds every 48 h. Cell confluence was measured every 24 h using a Celigo Cytometer (Cyntellect Inc., USA). For colony formation assay with irradiation, cells were transfected with siRNA as mentioned above. After 48 h of transfection, cells were seeded (720–3000 cells/well) and irradiated with a single dose of 1, 2, 4, 6, and 8 Gy (Gulmay Medical, Camberley, UK), and a standard colony-forming assay was performed to determine the respective surviving fractions (SF). After 19 days, the colonies were fixed with 70% ethanol for 20 min and stained with Mayer’s hemalaun for 5 min and colonies (> 50 cells) were counted and SF (*n* = 3) were represented in the graph [35]. Data were normalized to plating efficiency for each condition and are represented as mean (*n* = 3) ± SD. *P* values were calculated by ANOVA method (*P ≤ 0.05, **P ≤ 0.01, and ***P ≤ 0.001).

### Chromatin fractionation

Chromatin fractionation was performed as previously described [34]. Briefly, cells were re-suspended in lysis buffer [10 mM HEPES (pH 7.9), 10 mM KCl, 1.5 mM MgCl_2_, 0.34 M sucrose, 10% glycerol, 0.1% Triton X-100, 1 mM DTT, 1 mM Pefabloc, 1 ng/μL Aprotinin/Leupeptin, 10 mM β-glycerophosphate, and 1 mM N-ethylmaleimide] and nuclei were separated by centrifugation and then lysed in nuclear lysis buffer [3 mM EDTA, 0.2 mM EGTA, 1 mM DTT, and protease inhibitors] for 30 min on ice. Soluble chromatin fractions were separated by centrifugation and analyzed by SDS-PAGE electrophoresis.

### Proximity ligation assay

Proximity ligation assay (PLA) was performed using the Duolink® In Situ Red Starter Kit Mouse/Rabbit (DUO92101, Sigma-Aldrich, USA) according to the manufacturer’s instructions. The cells were processed similar to immunofluorescence staining with specific primary antibodies. After that, cells were incubated with secondary antibodies conjugated to PLA probes followed by ligation and amplification according to the manufacturer’s instructions. The images were acquired using a Zeiss LSM 510 Meta confocal microscope using 25X or 63X oil immersion lens.

### Statistical significance

Experiments were performed in biological triplicates (*n* = 3) and the mean values are represented in the graph. The statistical significance (standard deviation, ± SD) was calculated and *p* values were calculated using ANOVA represented as ns—*p* > 0.05, *p ≤ 0.05, **p ≤ 0.01, ***p ≤ 0.001.

## Additional files

Additional file 1: **Figure S1.** Knockdown of DOT1L leads to decreased H3K79 methylation. a SW837 cells were transfected with either control or individual or smart pool (4 siRNAs) DOT1L siRNAs (SP). After 72 h of transfection, relative DOT1L mRNA expression levels in control or DOT1L siRNA-transfected cells were measured using qPCR. b Similar to a, SW837 cells were transfected and total protein lysate was immunoblotted with DOT1L and H3K79me3 antibodies. H3 and HSC70 were used as loading controls. M, G and N were used as 3 independent controls (M – mock, G – GL2 Duplex non-targeting siRNA, N – siGENOME non-targeting siRNA). c Similar to Fig. 1b, U2OS cells were transfected with siDOT1L (smart pool) and treated with NCS for indicated time points and processed for immunofluorescence and stained with pKAP1 antibody. d and e Quantification of Western blot data from Fig. 1d for represented proteins. f SW837 cells after the indicated time points following NCS (100 ng/ml) treatment. PLA assay was performed as described in Fig. 1f using γH2AX and 53BP1 antibodies as a positive control and BSA as a negative control (scale bar – 10 μM). f Whole cell extracts from U2OS cells transfected similar to Fig. 1a were analyzed by Western blot for indicated proteins. (TIF 3056 kb)

Additional file 2: **Figure S2.** a HCT116 cells harboring a single copy of pHPRT-pDRGFP (HR substrate) were transfected with the indicated siRNAs and/or plasmid constructs as mentioned in the methods and proteins lysates were analyzed 48 h later by Western blot for the indicated proteins. b Whole cell extracts from U2OS cells were transfected similar to Fig. 1a and analyzed by Western blot for pRAD51. c Cell cycle analysis using SW837 cells transfected with either mock or DOT1L siRNA (smart pool) and after 48 h of transfection cells were treated with NCS for the indicated time points and processed for propidium iodide (PI) based flow cytometry as mentioned in the methods. The percentages of cells in each phase of cell cycle are represented in the graph (*n* = 3, ±SD). (TIF 1026 kb)

Additional file 3: **Figure S3.** a SW837 cells were treated either with DMSO or the DOT1L inhibitor EPZ5676 (1 μM) for either 3 h (acute) or 72 h (prolonged) followed by NCS (100 ng/ml) treatment for the indicated time points. Total protein lysates were analyzed by Western blot analysis for the indicated proteins. b Similar to a SW837 cells were treated either with DMSO or DOT1L inhibitor SGC094a (100 nM) followed by treatment with NCS. Total protein lysates were analyzed by Western blot for the indicated proteins. (TIF 572 kb)

Additional file 4: Supplementary Methods. (DOCX 25 kb)

## Abbreviations

ATM: Ataxia telangiectasia mutated kinase
CRC: Colorectal cancer
CtIP: CtBP-interacting protein
PARP: Poly (adenosine diphosphate [ADP]) ribose polymerase
RPA: Replication protein A
