# Supplementary figures and images for: The histone methyltransferase DOT1L is required for proper DNA damage response, DNA repair, and modulates chemotherapy responsiveness
Source: Clin Epigenetics. 2019 Jan 7;11:4. doi: 10.1186/s13148-018-0601-1 (PMC6323691; doi:10.1186/s13148-018-0601-1)

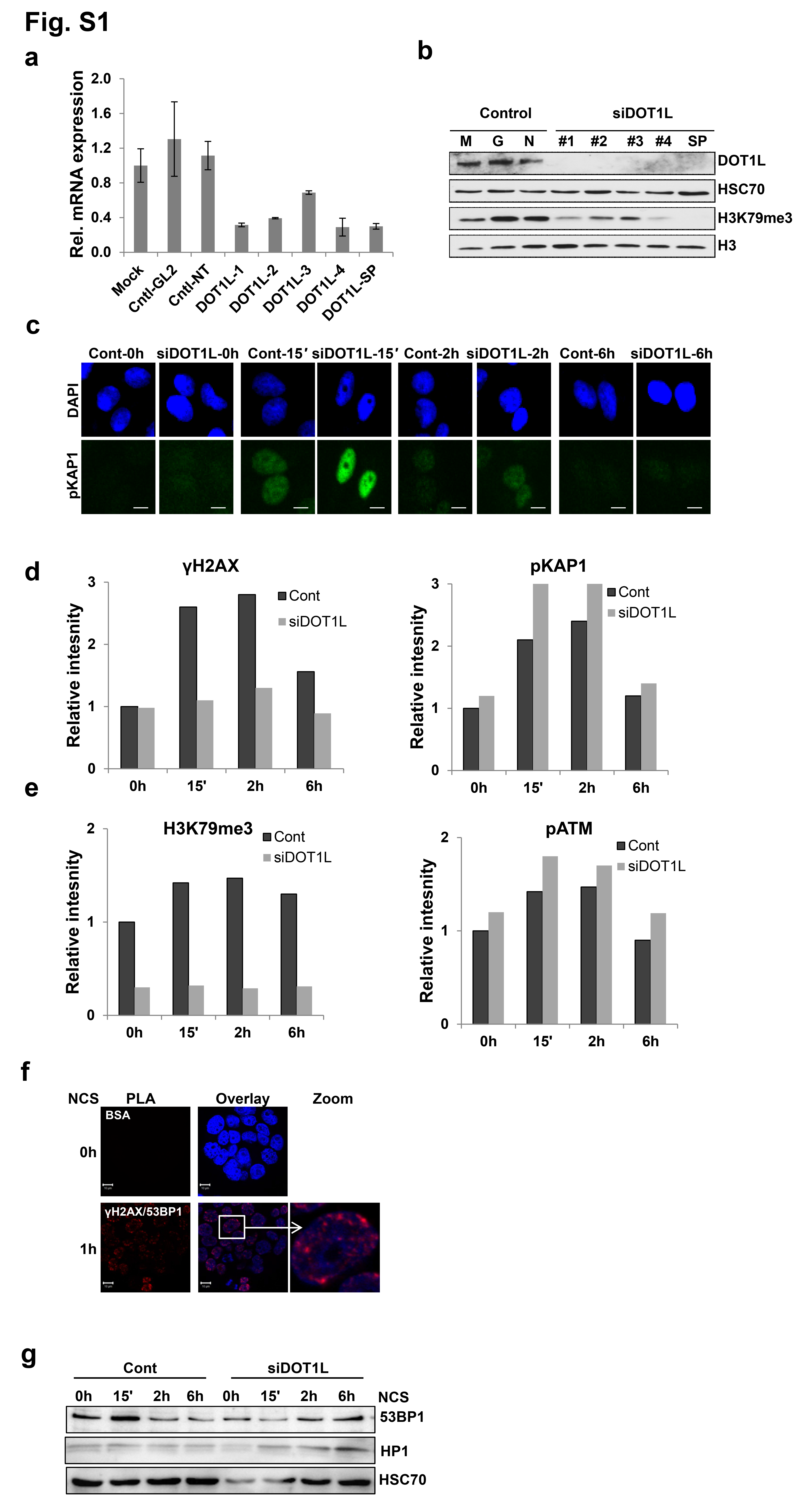

Supplement: Supplementary file 1 — Figure S1. Knockdown of DOT1L leads to decreased H3K79 methylation. a SW837 cells were transfected with either control or individual or smart pool (4 siRNAs) DOT1L siRNAs (SP). After 72 h of transfection, relative DOT1L mRNA expression levels in control or DOT1L siRNA-transfected cells were measured using qPCR. b Similar to a, SW837 cells were transfected and total protein lysate was immunoblotted with DOT1L and H3K79me3 antibodies. H3 and HSC70 were used as loading controls. M, G and N were used as 3 independent controls (M – mock, G – GL2 Duplex non-targeting siRNA, N – siGENOME non-targeting siRNA). c Similar to Fig. 1b, U2OS cells were transfected with siDOT1L (smart pool) and treated with NCS for indicated time points and processed for immunofluorescence and stained with pKAP1 antibody. d and e Quantification of Western blot data from Fig. 1d for represented proteins. f SW837 cells after the indicated time points following NCS (100 ng/ml) treatment. PLA assay was performed as described in Fig. 1f using γH2AX and 53BP1 antibodies as a positive control and BSA as a negative control (scale bar – 10 μM). f Whole cell extracts from U2OS cells transfected similar to Fig. 1a were analyzed by Western blot for indicated proteins. (TIF 3056 kb) [file 13148_2018_601_MOESM1_ESM.tif]

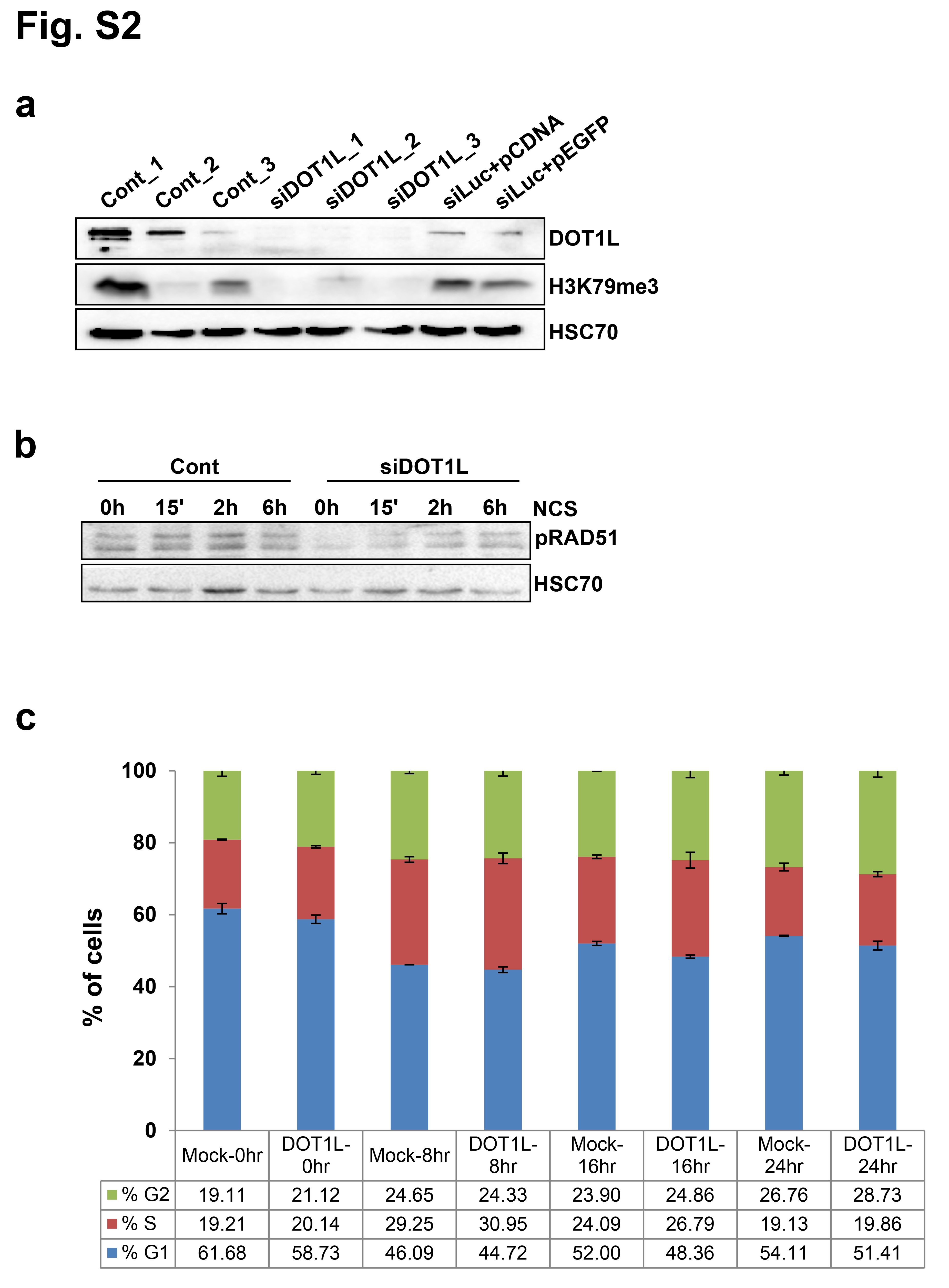

Supplement: Supplementary file 2 — Figure S2. a HCT116 cells harboring a single copy of pHPRT-pDRGFP (HR substrate) were transfected with the indicated siRNAs and/or plasmid constructs as mentioned in the methods and proteins lysates were analyzed 48 h later by Western blot for the indicated proteins. b Whole cell extracts from U2OS cells were transfected similar to Fig. 1a and analyzed by Western blot for pRAD51. c Cell cycle analysis using SW837 cells transfected with either mock or DOT1L siRNA (smart pool) and after 48 h of transfection cells were treated with NCS for the indicated time points and processed for propidium iodide (PI) based flow cytometry as mentioned in the methods. The percentages of cells in each phase of cell cycle are represented in the graph (n = 3, ±SD). (TIF 1026 kb) [file 13148_2018_601_MOESM2_ESM.tif]

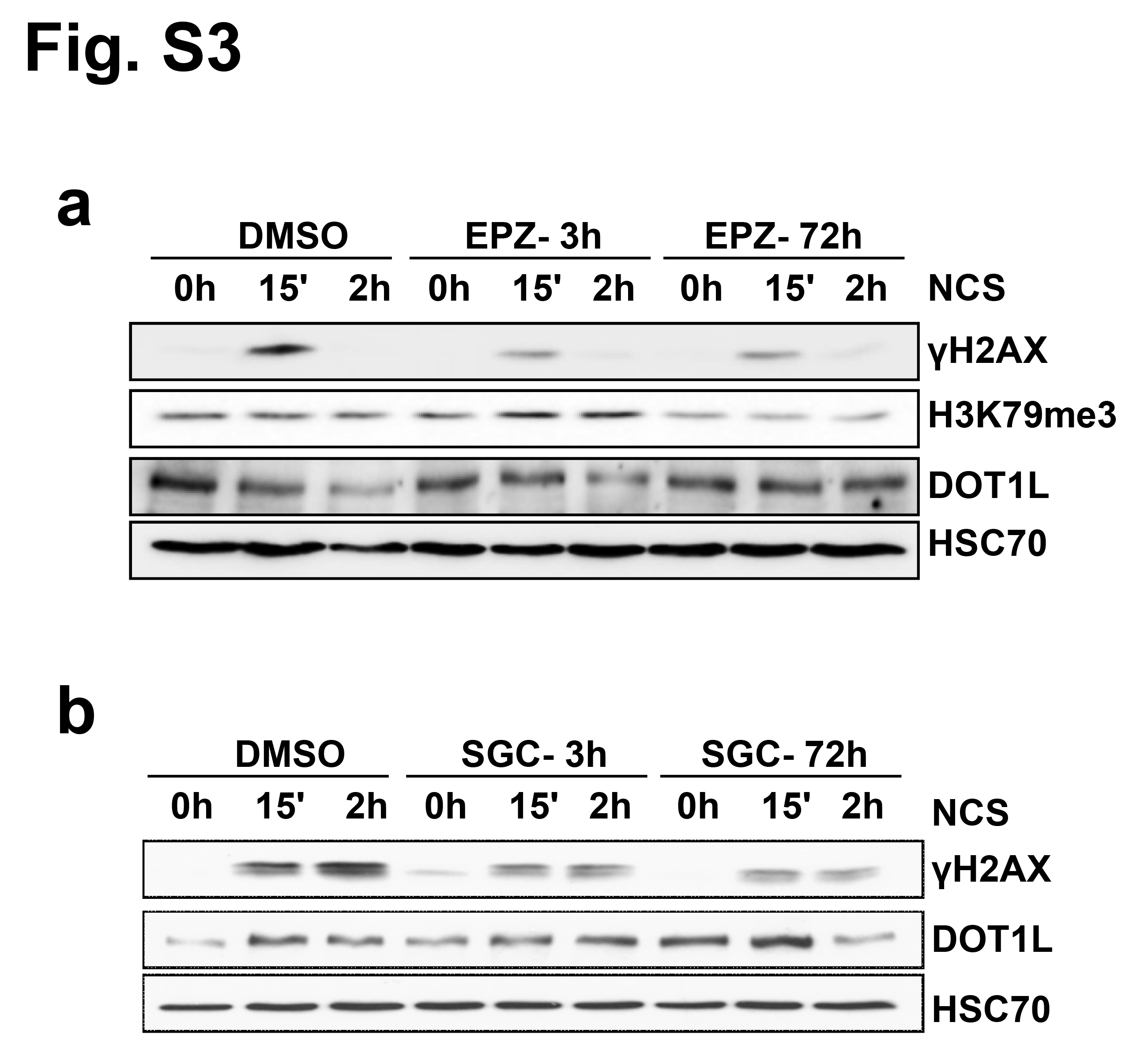

Supplement: Supplementary file 3 — Figure S3. a SW837 cells were treated either with DMSO or the DOT1L inhibitor EPZ5676 (1 μM) for either 3 h (acute) or 72 h (prolonged) followed by NCS (100 ng/ml) treatment for the indicated time points. Total protein lysates were analyzed by Western blot analysis for the indicated proteins. b Similar to a SW837 cells were treated either with DMSO or DOT1L inhibitor SGC094a (100 nM) followed by treatment with NCS. Total protein lysates were analyzed by Western blot for the indicated proteins. (TIF 572 kb) [file 13148_2018_601_MOESM3_ESM.tif]
