# Supplementary material for: The histone methyltransferase DOT1L is required for proper DNA damage response, DNA repair, and modulates chemotherapy responsiveness
Source: Clin Epigenetics. 2019 Jan 7;11:4. doi: 10.1186/s13148-018-0601-1 (PMC6323691; doi:10.1186/s13148-018-0601-1)
Supplement: Supplementary file 4 — Supplementary Methods. (DOCX 25 kb) [file 13148_2018_601_MOESM4_ESM.docx]

**Supplementary Methods**

**RNA isolation and quantitative real-time PCR (qRT-PCR)**

Total RNA was isolated from cells using TRIzol® Reagent (Invitrogen, USA) according to the manufacturer’s instructions. For the cDNA preparation 1 µg of total RNA was reversed transcribed using random nonamer primers. For checking the knockdown efficiency of DOT1L with individual and SmartPool (4) of siRNAs, qRT-PCR was performed with primers indicated in the table 2. For gene expression data is normalized to HNRNPK and represented as fold change relative to mock transfection.

**Fluorescence-Activated Cell Sorting (FACS)**

SW837 cells were transfected with DOT1L siRNA or mock. After 48 h of transfection cells were treated with 100ng/ml NCS for the indicated times. Cells were trypsinized and washed with ice cold PBS++ and resuspended in PBS++ and ethanol (final concentration 70%) and incubated overnight at 4°C. Cells were rehydrated with PBS++ and treated with RNAse A for 30 min at 37°C. The cells were labelled with propidium iodide according to the manufacturer’s instruction and analyzed by FACS.
